# Portal vein thrombosis in patients with hepatocellular carcinoma and early cirrhosis—prevalence and risk factors

**DOI:** 10.3332/ecancer.2023.1581

**Published:** 2023-07-26

**Authors:** Muhammad Tayyab Ul-Hasan Siddiqui, Ghulam Fareed, Muhammad Rizwan Khan, Amna Riaz, Saeed Sadiq Hamid

**Affiliations:** 1 Department of Surgery, Patel Hospital, Karachi 75300, Pakistan; 2 Department of Medicine, Kulsum International Hospital, Islamabad 44000, Pakistan; 3 Department of Surgery, Aga Khan University Hospital, Karachi 74000, Pakistan; 4 Department of Medicine, Aga Khan University Hospital, Karachi 74000, Pakistan

**Keywords:** hepatocellular carcinoma, portal vein thrombosis, Child–Pugh class, risk factors

## Abstract

**Introduction:**

Hepatocellular carcinoma (HCC) is frequently associated with portal vein thrombosis (PVT) with prevalence ranging from 25% to 50%. PVT is associated with poor prognosis, limiting the available therapeutic options for these patients. Our objective was to determine the prevalence and risk factors for PVT in patients with HCC.

**Method:**

A retrospective analysis was performed on the prospectively collected data from January 2018 to March 2020. All patients with HCC discussed in our weekly multidisciplinary liver clinic were reviewed. Multivariate analysis was done to identify the independent risk factors for PVT in HCC patients. A *p*-value of <0.05 was considered significant.

**Result:**

Of 316 patients, the prevalence of PVT was 31% (*n* = 98). Larger tumour size (*p* < 0.001), raised Alpha Fetoprotein (AFP) level (*p* = 0.036) and higher Child–Pugh class (*p* = 0.008) were significantly associated with PVT. In 216 patients with preserved liver function (Child–Pugh class A), PVT was seen in 53 (24.5%) patients. Large tumour size (*p* < 0.001) and higher AFP levels (*p* = 0.021) were independent risk factors.

**Conclusion:**

Overall prevalence of PVT in HCC was 31% whereas 24.5% in patients with early cirrhosis (Child–Pugh class A). We identified various risk factors associated with PVT in our local population, highlighting the importance of early and regular screening of cirrhotic patients including Child–Pugh class A.

## Introduction

Hepatocellular carcinoma (HCC) is the sixth most commonly diagnosed cancer and the third leading cause of cancer-related deaths worldwide [1]. Hepatitis C Virus (HCV) infection has been reported as the most common risk factor (60%–70%) for HCC in Pakistan [2]. Recent emerging data have shown that fatty liver disease is one of the leading causes of liver-related health problems. It is estimated that globally, every third adult is suffering from fatty liver disease and it is equally important and problematic in children too [3, 4]. Fatty liver disease has an established association with other medical conditions including metabolic syndrome, type-2 diabetes mellitus, obesity and cardiovascular diseases [5–7]. As its burden has increased over time, the preparedness has also increased, but South Asia is still down on the list [8]. In Pakistan, the prevalence of fatty liver disease has been increasing, and it is estimated to be 14%–41% in various studies conducted on the local population [9]. Unfortunately, the exact incidence and prevalence of HCC are not known in Pakistan due to a lack of population-based studies. Pakistan is considered to be in the intermediate zone for HCC prevalence, and it is considered to be one of the big threats in Pakistan [10, 11]. Hospital-based data show that hepatobiliary cancers are the most common malignancies in males over 18 years of age in our population [12]. It is important to note that the majority of HCC patients in Pakistan have advanced disease at the time of diagnosis and only less than a third are eligible for definitive treatment [13]. This is partly because of our weak health care system as well as a variable clinical spectrum of disease behaviour.

The risk of thrombosis in patients with malignancy has been reported to be nine times higher than those without malignancy, and it is one of the leading causes of death in cancer patients [14, 15]. Likewise, HCC has a known predilection for macrovascular invasion. Portal vein thrombus (PVT) is a relatively common presentation, observed in 25%–50% of patients with HCC in the reported literature [16]. PVT when associated with HCC is considered to be an advanced stage of the disease with a poor prognosis since only limited therapeutic options are available [16].

The multidisciplinary team (MDT) approach provides a timely, individualised treatment plan for patients with HCC and improves the patient’s survival [17]. In advanced HCC, especially those with vascular invasion, these MDTs provide maximum benefit to the patient by providing specifically tailored management plans after all relevant teams input [17, 18]. Weekly multidisciplinary liver clinics at Aga Khan University Hospital were started at the end of 2017 where all patients with HCC are discussed. During these years the group observed more than usual frequency of advanced stage of HCC in an otherwise well-preserved liver (Child–Pugh Class A). Due to very limited and variable local literature on this topic, we designed a study to determine the prevalence and risk factors of PVT in patients with HCC, especially with preserved liver function.

## Methodology

We conducted an analytical cross sectional study on prospectively collected data from January 2018 to March 2020 for all patients discussed in the multidisciplinary liver tumour board at Aga Khan University Hospital. These meetings were named multidisciplinary liver cancer clinics because the meetings not only included detailed clinical discussions amongst subject specialists leading to patient-specific clinical decisions but also included the active participation of the patients and their families in the final decision-making process.

All adult patients with HCC who were discussed in the liver clinic meetings during the specified period were included (*n* = 406). Ninety patients were excluded for various reasons, mainly due to incomplete records, and 316 patients were included in the study. Computerized tomography (CT) scan (triphasic) was the radiological modality used on all patients to identify the presence of PVT. Data were collected using a structured questionnaire including information on demographics, comorbidities, performance status (EOG), laboratory tests (complete blood counts, liver function tests, coagulation tests, albumin level, tumour marker alpha fetoprotein (AFP) and hepatitis serology), and radiological findings specify the presence of thrombosis in the portal vein. Child–Pugh Score and Model for End-Stage Liver Disease (MELD) score were calculated for the estimation of underlying liver function. Barcelona Clinic liver cancer (BCLC) staging system was utilised to stage HCC burden.

Data were analysed using the Statistical Package for Social Sciences version 22. Quantitative variables are reported as mean ± SD or median with inter-quartile range (IQR), while qualitative variables are reported as frequency and percentages. Receiver operating characteristic (ROC) curve analysis was performed to identify the cut-off for the size of tumour on available data. Multivariate analysis was done to identify the independent clinical, laboratory and radiological risk factors for PVT in patients with HCC. A *p*-value of <0.05 was considered significant. Institutional Ethical Review Committee approval was taken before the start of the study.

## Results

We analysed 316 patients with the diagnosis of HCC. The mean age of our population was 60 ± 11 years and the majority were males (*n* = 245, 77%) with good functional class i.e., Eastern Cooperative Oncology Group (ECOG) 0 and 1 (*n* = 266, 84%). Approximately 50% had associated comorbid conditions, whereas 76 (24%) patients had more than one comorbidity. Hepatitis C was the most frequent risk factor for HCC (*n* = 187, 59%) followed by hepatitis B (*n* = 37, 11%), although significant cases (*n* = 78, 24%) had unknown underlying aetiology due to lack of liver biopsy, possibly nonalcoholic steatohepatitis (NASH). More than 90% of the patients presented with clinical symptoms, the most common being abdominal pain (*n* = 151, 52%). A vast majority of the patients had preserved liver function, and Child–Pugh class A was seen in 216 (68.4%) patients. The median size of tumour was 5.4 cm (IQR 2.6–10 cm). We found that cut-off tumour size for our population is 5.9 cm above which the chances of PVT are higher, with a sensitivity of 71.4% and specificity of 69.5% with an area under the curve of 71.5%. Table 1 demonstrates the patient- and tumour-related characteristics.

PVT was diagnosed in 98 patients (31%). The site of thrombosis included main portal vein in 38 (12%) patients and branch portal vein in 58 (18.4%) patients; while 2 (0.6%) patients had thrombosis in other systemic vein along with PVT. Univariate regression analysis revealed a number of factors associated with the presence of PVT in patients with HCC as outlined in Table 2. On multivariate analysis, larger tumour size, raised AFP level and higher Child–Pugh class continued to be independently associated with PVT in HCC.

Table 3 shows the distribution of PVT as per Child–Pugh classification. The association of PVT increased significantly (*p* = 0.000) with worsening liver function ranging from 24% in class A and 50% in class C. Child–Pugh Class C had higher chances of main PVT whereas Class A had higher chances of having branch PVT. Factors associated with PVT in patients with preserved liver function (Child–Pugh class A) were identified using univariate and multivariate regression analysis (Table 4). Large tumour size (*p* = 0.000) and high AFP levels (0.012) were independently associated with PVT in HCC patients with preserved liver function.

## Discussion

HCC is a common malignancy seen in the Pakistani population with a prevalence of 3.7%–16% [11]. Portal vein tumour thrombosis in patients with HCC has been variably reported in the literature with a frequency of 10%–40%. In our study, the prevalence of portal vein tumour thrombus was 31% (*n* = 98), which seems to fall within the reported range. Similarly, tumour thrombosis in patients with preserved liver function is also not uncommon and the reported incidence in Child–Pugh class A cirrhotic with HCC in the Western population is 28.1% [19], akin to our observed prevalence rate of 24.5%. The majority of patients included in our study had cirrhosis due to underlying HCV infection, a finding similar to the previously published data on HCC [2, 10, 11, 20–22].

Traditionally, direct invasion of the venous wall with HCC is a well-known mechanism for PVT but in reality, it is much more complex and tumour microenvironment in the background of cirrhosis plays a major role [23]. Tumour cells have specific properties that enhance the prothrombotic state. These cells can directly interact with blood cells like platelets and endothelial cells, involved in the production and release of procoagulants and inflammatory cytokines, hence promoting hypercoagulable state for PVT [24]. Other risk factors for PVT include decreased portal flow velocity, wide portal vein, large spleen size, thrombocytopenia and advanced cirrhosis [25]. Yan *et al* [26], recently published an article on the association of splenic vein and superior mesenteric vein angulation with the thedevelopment of PVT.

Incidental finding of PVT in HCC patients with relatively preserved liver function (Child–Pugh class-A) significantly impacts their overall management and prognosis. PVT in HCC has a major impact on treatment decision, whether to go for aggressive curative intend treatment or limit oneself to palliation only [23]. BCLC staging system is widely used for categorising HCC patients depending upon tumour burden (size and the number of lesions), patient’s ECOG status, overall hepatic functions and presence and absence of vascular invasion including PVT. Liver cancer study group in Japan proposed a specific classification system for PVT in HCC, this helps further to tailor a specific management plan for each patient [23].

In our study, we identified multiple risk factors which were significantly associated with PVT. We found worsening liver function as reflected by a high Child–Pugh score was significantly associated with the development of PVT with HCC. This finding was in accordance with the available literature [21, 27]. The size of the tumour was found to be an independent factor for PVT in HCC, this is supported by both national and international literature [21, 27, 28]. Similar to ours, Shabana *et al* [27] identified a 5.9 cm cut-off for tumour size above which the risk of having PVT in HCC is higher with a sensitivity of 64.5%, specificity of 72.1% and area under the ROC curve (AUC) of 70.5%. AFP levels are the most commonly used tumour marker for HCC. It has a significant role in screening, diagnosis, monitoring treatment response, aggressiveness of tumour and prognosis [29]. AFP levels are found to be independently associated with the aggressiveness of HCC inform of thrombosis [27]. Likewise, we found high serum AFP levels to be an independent risk factor for PVT in HCC patients irrespective of liver function status. Certain risk factors that were found to be significant in other studies like advanced age, gender and decreased albumin levels were not found significant in our study [21, 30]. Viral versus non-viral aetiology for cirrhosis was found to be insignificant for tumour invasion, which is comparable to available literature [21, 31]. More than 90% of our patients present with symptoms, predominantly abdominal pain, jaundice and ascites. Less than 10% were those, who were picked up by routine screening, highlighting lack of screening is a major risk factor in these high-risk cirrhotic patients.

Our study is a single institution experience but with referrals from all over the country and a diverse population. There are certain limitations of our study including male predominance which makes it less generalisable although it is already established that men have higher rates of liver fibrosis, cirrhosis and HCC. A second limitation is that fatty liver disease would be the most common cause for cirrhosis and HCC globally, in our population liver biopsy was not done in all patients to confirm the aetiology. HCV was the most common aetiology for HCC in our population based on available records. A third limitation of our study is the lack of data on survival outcomes in our population, the reason being that the patients received treatment at different institutions following the recommendation of our multidisciplinary liver cancer clinics. Despite the limitations, our study would help us identify risk factors associated with PVT with HCC in early cirrhotics. Multicentre studies, especially including institutions with liver transplantation facility should focus on developing therapeutic strategies in this special group of patients.

## Conclusion

HCC is a major health concern in Pakistan. In our study, almost every third patient of HCC had vascular invasion like PVT and every fourth patient with early cirrhosis developed HCC with vascular invasion. We identified multiple risk factors in early cirrhosis that were associated with vascular invasion including raised AFPs, greater tumour size and lack of surveillance for HCC in cirrhosis. HCC surveillance needs to be improved in early cirrhosis, and multi-centric prospective studies are required in our population for identifying more risk factors.

## Conflicts of interest

None of the authors have any conflict of interest or financial affiliation to disclose.

## Funding statement

The authors did not receive any grant from any funding agency to conduct this study.

## Figures and Tables

**Table 1.** Demographics and disease characteristics of patients with HCC.

Characteristics			Frequency	Percent
Total (*n*)			316	
Age (years)	Median (IQR)		60 (54–67)	
Gender	Male		245	77
	Female		71	23
Comorbidities	None		162	50
	Multiple		76	24
	Diabetes		34	11
	Hypertension		34	11
	Other		10	3.2
Underlying risk factors for HCC	Hepatitis C		187	59
	Hepatitis B		37	11
	Other non-viral causes		19	6
	Unknown (likely non alcoholic fatty liver disease)		78	24
Performance status: ECOG	0		136	43
	1		130	41
	2		36	11.4
	3		13	4
	4		1	0.6
Mode of presentation	Abdominal pain		151	52
	Jaundice		8	2.5
	Weakness and weight loss		39	12.34
	Ascites		59	18.7
	Upper GI bleed		38	12
	Encephalopathy		11	3.5
	Other symptoms		5	1.6
	Incidental diagnosis		20	7
	Diagnosis on screening		6	2
Laboratory values	Median platelet per cubic millimeter (Median/IQR)		144 (91–214)	
	Total bilirubin g/dL (Median/range)		1 (0.6–1.7)	
	International normalised ratio – INR (Median/range)		1.1 (1.05–1.3)	
	Serum albumin g/dl (Median + SD)		3.4 (2.9 + 3.9)	
	Alpha fetoprotein – AFP ng/mL (Median/IQR)		42 (6–627)	
Radiological findings	Median tumour size in cm (IQR) (*n* = 281)		5.4 (2.6–10)	
	No. of lesions (*n* = 303)	1	166	54.7
		2–3	64	21
		>3	73	24
	Underlying cirrhosis		270	85.4
	Extrahepatic involvement of HCC		39	12.3
	Presence of PVT		98	31
Child–Pugh class	A		216	68.4
	B		76	24
	C		24	7.6
MELD score	6–16		289	91.4
	17–40		21	6.6
BCLC staging	0		17	5.4
	A		91	28.8
	B		64	20.3
	C		123	39
	D		10	3.2

**Table 2.** Factors predictive of PVT in HCC.

PVT in HCC		
Risk factors	OR (95% Confidence interval)	*p* value
Univariate analysis		
Pain	0.615 (0.381–0.995)	0.047
Jaundice	0.142 (0.028–0.717)	0.018
Liver *decompensation*	0.534 (0.327–0.871)	0.012
Raised bilirubin level (>1.2)	2.062 (1.261–3.373)	0.004
Raised AFP level (>40)	2.247 (1.358–3.719)	0.002
Tumour size (>5.9 cm)	5.708 (3.253–10.016)	0.000
Ascites	0.204 (0.065–0.639)	0.006
MELD score	0.928 (0.882–0.976)	0.004
CHILD–PUGH (A versus BC)	2.516 (1.524–4.154)	0.000
Multivariate analysis		
Tumour size (>5.9 cm)	4.99 (2.79–8.925)	0.000
Raised AFP level (>40)	1.935 (1.077–3.476)	0.027
CHILD–PUGH (A versus BC)	2.122 (1.168–3.857)	0.014

**Table 3.** Distribution of PVT according to Child–Pugh class.

Thrombosis status	Child A (n = 216)	Child B (n = 76)	Child C (n = 24)	Total (n = 316)
PVT (present)	53 (24.5%)	33 (43.4%)	12 (50%)	98 (31%)
Branch PV	37 (17.12%)	17 (22.36%)	4 (16.66%)	58 (18.4%)
Main PV	15 (6.9%)	15 (19.73%)	8 (33.33%)	38 (12%)
IVC/SMV	1 (0.46%)	1 (1.3%)	0	2 (0.6%)

**Table 4.** Factors predictive of PVT in HCC patients with preserved liver function (CHILD A *n* = 216).

PVT in HCC with Child A class		
Factors	OR (95% Confidence Interval)	*p* value
Univariate analysis		
Pain	0.495 (0.264–0.929)	0.029
Tumour size (>5.9 cm)	7.879 (3.661–16.955)	0.000
Raised AFP level (>40)	2.607 (1.187–5.728)	0.017
Multivariate analysis		
Tumour size (>5.9 cm)	7.408 (3.393–16.176)	0.000
Raised AFP level (>40)	2.642 (1.236–5.649)	0.012
